# A total productive maintenance & reliability framework for an active pharmaceutical ingredient plant utilising design for Lean Six Sigma

**DOI:** 10.1016/j.heliyon.2023.e20516

**Published:** 2023-10-17

**Authors:** Noel Shannon, Anna Trubetskaya, Javed Iqbal, Olivia McDermott

**Affiliations:** a Department of Engineering, University of Limerick, Limerick, Ireland; b Department of Biosciences and Aquaculture, Nord University, Steinkjer, Norway; c Department of Chemical Sciences, School of Natural Sciences, University of Limerick, Limerick, Ireland; d College of Science and Engineering, University of Galway, Galway, Ireland

**Keywords:** Manufacturing excellence, Total productive maintenance, Autonomous maintenance, Operator care, Overall equipment effectiveness (OEE), Lean Six Sigma, Maintenance strategy

## Abstract

This research investigates the requirement for and relationship and implementation of a total productive maintenance (TPM) and Reliability Centred Maintenance (RCM) strategy within an Active Pharmaceutical Ingredient (API) Plant. This research aimed to study the tools and techniques of TPM and Reliability Engineering and then deploy a designed model to an API plant. A case study involving Design for Lean Six Sigma phases of Define, Measure, Analyse, Design, and Verify was utilised to build an API site TPM pilot program. Data was collected using interviews across Company 'X's local and Global Engineering teams. Process runtime, downtime and plant availability metrics were compiled and a new design for Total Productive Maintenance and Reliability was proposed and verified. A maintenance framework was designed to optimally incorporate Total Productive Maintenance, Reliability and Operational Excellence with an emphasis on Overall Equipment Efficiency (OEE) realizing a 33 % reduction in planned maintenance activities, a 70 % reduction in Corrective Maintenance, Cleaning for Maintenance was reduced by 50 %, the pilot maintenance area of the centrifuge has its OEE increased by 20 % and plant availability increased by two hundred and 6 h. This research highlights the importance of Total Productive Maintenance as a key component of an effective maintenance strategy and its potential to transform maintenance practices. Based on this research and results, TPM is recommended to be applied to any API manufacturing organization. A limitation of the study is that it is a single-site case study. The novelty of this research is based upon the emphasis on Reliability Engineering to remove non-value add Maintenance time from the manufacturing schedule. The Total Productive Maintenance & Reliability model designed and implemented in this research is unique in the literature and can be leveraged by engineering professionals and academics to understand the benefits of TPM.

## 1 Introduction

Manufacturing organizations strive to operate competitively with high productivity, timely delivery, and enhanced quality and can contribute to this by reducing line downtime. The interaction between Maintenance and Production to reduce line downtime has been very important to manufacturing organizations as a good maintenance process, or policy can enhance productivity [1]. A poor and insufficient maintenance system can seriously impact profitability, business continuity and the company's survival [2].

Maintenance is an activity that involves the inspection and repair of a machine at regular intervals to extend its useable life. Maintenance is regarded as a value-added activity rather than a necessary evil for the budget [3]. It is described as the operation required to keep a facility in “as built” condition, allowing it to retain its original productive potential [4]. Dhillon [5] classified Maintenance into three types, preventive, corrective or reliability-centred.

Total Productive Maintenance (TPM) is a comprehensive maintenance strategy that aims to improve equipment reliability, reduce downtime, and increase overall productivity in manufacturing industries [6]. TPM is based on the principle that every operator and maintenance employee is responsible for equipment maintenance [7]. TPM enables collaboration as the operator can help assist with routine and simple maintenance activities, while maintenance employees focus on more complex maintenance tasks [8]. TPM was practiced in Japan in the 1970s and was first introduced as a maintenance concept by Seiichi Nakajima, who, with the Japanese Institute of Plant Maintenance (JIPM), formed preventative maintenance (PM) research group [9]. JIPM described TPM as a system of Maintenance covering the total life of equipment in every area of the organization, including planning, manufacturing, and Maintenance [10]. TPM is recognized as a key pillar or tool of Lean and Lean Six Sigma (LSS) Manufacturing as it focuses on optimizing equipment and line productivity and thus aiding waste reduction and is an important pillar in continuous improvement programs [11,12]. TPM has also been linked to Lean-Green and sustainability benefits via its reduced material use, energy usage, toxic emission reduction and overall waste reduction [13].

Operating Equipment Effectiveness (OEE) is widely utilised as a TPM metric. OEE was created to provide a useful indication for recognizing and quantifying inefficiencies in industrial equipment, which are categorized into three types of losses: time losses, speed losses, and quality losses [14]. An OEE percentage of 85 % is best in class as a benchmark across all manufacturing industries [15], and research has demonstrated key correlations between TPM, TQM and Operational Excellence [16,17]*.* The University of St. Gallen has carried out several studies on the Pharma industry productivity and operational excellence from 2005 onwards and has demonstrated that improving productivity has become a huge focus of the Pharma industry due to rising cost pressures [18,19]. However, during this period, there has been no real improvement in pharmaceutical industry productivity [20].

This research occurs within an active pharmaceutical ingredient (API) plant where the OEE metric was demonstrating losses in productivity. Production time was being lost due to high maintenance time. The regulated nature of the pharmaceutical industry requires that due to the nature of ‘invasive’ maintenance tasks, there is a requirement for pre- and post-maintenance cleaning [21,22]. This cleaning would extend the time the equipment is unavailable to Production, so equipment availability is impacted beyond the maintenance task duration. The regulatory requirements in Active Pharmaceutical Ingredients product manufacture prescribed by different government jurisdictions can affect productivity due to increased cleaning, inspection and testing requirements at various points [21,22]. Periodic equipment cleaning is also required per the regulatory cleaning validation requirements onsite and inter-campaign cleaning (ICC) is required when changing from intermediate manufacture to API [23].

This research investigates implementing a total productive maintenance (TPM) program strategy for an Active Pharmaceutical Ingredient Plant (API) to resolve OEE, productivity and Maintenance downtime issues. While there are some examples of TPM implementation in the Pharmaceutical industry [17,24], the examples of TPM application in Pharmaceuticals are sparse within the literature. For example, Vaz et al. [25] carried out a study on the value of TPM in Portuguese companies and found that the Pharma sectors usage of TPM was ranked much lower (5th) behind other industries such as the food, heavy manufacturing and automotive industries usage of TPM. This research aims to develop a TPM framework tailored to an API plant and to understand if there is value in implementing TPM within the Pharma industry.

Thus, the research questions (RQs) for this study are:

RQ1 How can DFLSS methods be utilised to implement a customized TPM model to improve a Maintenance strategy at an API plant?

RQ2 Is there a relationship between TPM adoption at an API plant and Overall Equipment Efficiency (OEE)?

Section 2 outlines the literature review, Section 3 the methodology utilised and Sections 4, 5 present the Results and their discussion. Finally, Section 6 concludes the study.

## 2 Literature review

### 2.1 Types of maintenance

The area of Maintenance management still has great confusion regarding terminologies utilised for different types of Maintenance in industry sectors and across correlated literature [26]. Moubray [27] described an evolution of Maintenance as evolving from corrective Maintenance or “fix it when it breaks, to corrective/preventive Maintenance, which consists of corrective na scheduled overhauls. Subsequently, a third evolution consists of corrective/preventive/predictive, bringing in predictive condition monitoring techniques, failure and risk analysis, while finally, the final and current evolution involves Reliability-centred Maintenance (RCM) [27,28].

Preventive Maintenance consists of all activities performed on a planned, periodic, and defined schedule to keep an item/equipment in the stated functioning condition through inspecting and reconditioning [29]. Preventive maintenance practice has the main disadvantage of incurring unnecessary costs for replacing parts whose condition may be perfectly satisfactory [30]. Corrective Maintenance - unscheduled Maintenance or repair to return items/equipment to a defined state, performed because of flaws or failures observed by maintenance personnel or users [31]. Predictive Maintenance uses sophisticated measuring and signal processing tools to accurately predict and diagnose the status of items/equipment while they are in use [32].

Just-in-time (JIT) maintenance pertains to servicing a machinery component as necessary rather than on a predetermined plan. Plant operators would want to have confidence that the equipment needs repair before they schedule it because shutting down crucial equipment units for Maintenance is expensive and time-consuming [24].

Reliability Centred Maintenance (RCM) incorporates failure mode analysis and considers asset function and criticality to determine suitable Maintenance to detect and prevent the potential failure mode. This predictive maintenance methodology [33] relies on the Maintenance performed before the failure event. Risk-Based Maintenance (RBM) strategies are aligned with RCM. The diagnosis's accuracy defines its success, so prognostics are crucial to the success of condition-based Maintenance [34].

### 2.2 The value of TPM and RCM and its effects on TQM and operational excellence

Studies have revealed many signs, certain conditions or other indications that highlight, flag or precede almost all mechanical failures [35]. Due to this, many non‐destructive or Maintenance techniques have been developed to determine incipient failure [36]. Analyzing results periodically enable appropriate Maintenance and repair scheduling upon indicating poor equipment and machine condition [25,35]. The main objective of Maintenance is to prolong equipment and machine life and thus eliminate the requirement to conduct repairs when no failure has occurred or before a failure has occurred [30].

*Ben-Daya and Duffuaa* [3] defined maintenance tasks as “*operating in parallel with manufacturing, the operations unit work on producing the goods and maintenance unit work on delivering capacity* via *machine uptime*”. The capacity attainment delivered via an optimum maintenance program will also deliver the quality and quantity of the outputs.

Total Quality Management (TQM) is a philosophy for pursuing continuous improvement in customer satisfaction and profit that goes beyond just defect reduction and emphasizes cross-business process improvement [37,38]**.** In contrast, Maintenance can contribute towards quality improvement, with working and functioning equipment leading to fewer defects, less scrap and enhanced meeting of customer specifications [11].

However, Maintenance can contribute to additional quality risks and defects; for example, Maintenance, cleaning and storage conditions may foster opportunities for micro-organisms proliferation within processing equipment [39]. Due to the nature of ‘invasive’ maintenance tasks, there is a requirement for pre- and post-maintenance cleaning; this cleaning would extend the time the equipment is unavailable to Production, so equipment availability is impacted beyond the maintenance task duration. It is a regulatory requirement in Active Pharmaceutical Ingredients product manufacture [40] to validate and execute cleaning after maintenance interventions [41].

Total Productive Maintenance (TPM) is a productive maintenance concept designed to achieve comprehensive effectiveness of the production system by involving everyone within the organization (as aligned with TQM philosophy) [9]. TPM is composed of 3 concepts. The first “*Total*” means to involve all employees of the organization. Secondly, " *Productive*” means that the maintenance activities are completed in so far as possible without interrupting the daily productivity of the organization. Thirdly “*Maintenance*” refers to selecting the most appropriate type of Maintenance [42].

Regarding the “*Maintenance*” section of TPM, a study by Nakhjavani et al. [43] identified TPM opportunities as a potential 33 % reduction in processing waste via the introduction of the right maintenance framework. TPM connects maintenance and production roles by relaying the importance of operators maintaining the plant equipment [24]. The shared equipment responsibility encourages direct participation by plant equipment workers and can effectively increase productivity [16].

TPM is a crucial component of business success and therefore needs to be compulsory in designing a maintenance strategy or program [44]. The research noted that TQM and TPM need to be realised ahead of LEAN practices; this informs the project plan for implementation phases. A study of the application of Lean, Total Quality Control (TQC) and TPM paradigms demonstrated that all 3 concepts could improve manufacturing systems performance [37].

Within API manufacturing, the increased dependence on equipment has prompted the requirement to keep equipment in good condition [45]**.** Maintaining equipment in good condition requires a robust maintenance program with multiple manual and automated tasks [46]. This highlights the requirement for a maintenance cycle and a method to continuously improve the maintenance program by re-evaluation. ISO 14224 collected Reliability and Maintenance data for equipment in hazardous industries (API) [47]*.* As no specific international standards for maintenance API are available, this standard is used across the industry as a “reliability thesaurus” for implementing a maintenance process [48].

Deming identified that 85 % of failures result from problems with the system, not the people, and ineffective predictive and preventive Maintenance can be attributed to poor program management rather than a lack of technology [49]. Research acknowledges a requirement for Maintenance at an API plant driven by a robust strategy and system [32,50]. A key research methodology identified is selecting a model machine or area to pilot the TPM introduction [51]. A limitation of TPM is that it does not focus on the technical aspects of the asset or equipment throughout its Lifecycle [37]. So, it does not enable a forward view or plan for technology selection in managing and maximizing the asset's output. RCM programs' function needs consideration in a TPM deployment [52]. It was identified how maintenance systems in pharmaceutical companies as still reactive rather than foresighted [18]. They plan maintenance programs based on failure analyses. Conditioned-based maintenance practice, RCM and early resolution of repeat failures would enable a more proactive maintenance program and enable pharmaceutical companies to move forward with a robust maintenance strategy and practices [37]. Lastly, a robust TPM system incorporating RCM can enable a Lean process and enhance productivity and quality [53].

## 3 Methodology

### 3.1 Background

This study aims to be develop a Total Productive Maintenance & Reliability (TPMR) framework within an API plant in Ireland that was scaling up manufacturing of five commercially successfully APIs in animal health. The plant equipment and utilities are a hybrid of new and old technologies. The site is in operation since the 1960's, consists of 25 acres of plant and employs one hundred and fifteen people. The equipment reliability and plant availability requires a focused improvement to ensure that the site supports its commercial demand hence the deployment of TPMR.

The methodology chosen to develop and define the TPMR was Define for Lean Six Sigma (DFLSS). This structured problem solving methodology primarily enables the design of a new process or system for TPMR. DFLSS is a proven concept for new process design which incorporates the elimination of waste and reduction of variation of Lean principles and Six Sigma tools [54]. DFLSS with its structured methodology of *D*efine, *M*easure, *A*nalyse, *D*esign and *V*erify (DMADV) aids the development of the new maintenance resumes work and can be successfully applied to any new process, service or product design [55]. The literature review identified a gap in research connecting maintenance strategies with asset care [53,56]. This study aims to address this gap and incorporate equipment criticality analysis, failure modes identification and RCM techniques to strengthen the overall maintenance program and go beyond TPM. The study proposes a new Total Productive Maintenance & Reliability (TPMR) adopted as a pilot program at the API plant. DFLSS is utilised to design the TPMR process and testing the functionality and measure the success of the program within all project phases. The single case study approach demonstrates the capability of the case study to support empirical generalizations and aid practical research and generalisations [57,58].

### 3.2 Dflss & Dmadv

#### 3.2.1 Define

A pilot program team and project was launched supported by the site leadership. The selection of a model machine within the API facility allowed the piloting of the TPM introduction, identify barriers and enablers to the process while also allowing for a core group of resources to be trained on the principles of TPM [59].

A project team which was cross-functional was formed and included members from operations, engineering, quality and global Maintenance. In defining the project DFLSS tools such as a process map and SIPOC (Table 1), Voice of the Customer (VOC) and Critical to Quality (CTQ) was utilised to define and document the problem statement or required vision. The overall project requires support, and a communication plan will ensure stakeholders remain informed throughout the lifecycle so Leadership support can be maintained.

**Table 1:** Overview of the DMADV tools and methodology applied in this research (Source: Authors own).

DMADV	Deliverables	Tools Applied	Purpose of method
Define	Project Goals defined to include Scope of the project	1. Interviews 2. SIPOC 3. Project Charter	Voice of the customer Understand inputs and outputs Outline the scope, and deliverables of TPM
Measure	Collate baseline data to Understand the future state	4. Interviews/Downtime data Process maps	Ensure collective data is in scope Identify Waste
Analyse	Analysis of runtime, and downtime and process cleaning time	5. JMP and Excel	Analyse real data Analyse full impacts
Design	Design and Pilot TPM Program Build a robust process	6. Process Mapping 7. FMEA, RCM, CBM	Risk Rank, Failures identify Optimise Asset Care Plan
Verify	Launch of a TPM program and Collate feedback from resources	8. Data Analyse, Downtime and 5S. 9. KPI reporting	To ensure the value of TPM

#### 3.2.2 Measure

The measure phase of DFLSS or LSS is a key stage for gathering data and measuring performance terms of defining measures in this case, for TPM the measure is operating equipment effectiveness (OEE) which is a combination of equipment, resource, and management effectiveness. Variation within the current API operation and maintenance process needed identification and measurement in order to design a framework for TPM. In this measure phase, the team captured available data and observations about the equipment and maintenance processes and performances. This current state analysis would be supported via Pareto analysis where downtime and runtime could be statistically evaluated in terms of OEE. Maintenance control is vital in maintenance optimisation and so machine data capture and analysis can support the maintenance strategy [60].

Semi-Structured qualitative interviews as were voice of the customer (VOC) with managers within the global organisation who had implemented TPM was collated. A site understanding and TPM design requirements would be collated to build on with the local site program support***.*** This baseline analysis of the current process and the development of a TPM scoring for the process under evaluation was a key project success enabler.

#### 3.2.3 Analyse

A number of DFLSS tools were utilised as outlined in Table 2 to complete the analyse phase and aid the design of the new TPM framework. The outputs are described in more detail in Section 4.

**Table 2:** List of DFLSS tools utilised in the Analyse phase (Source: Authors own).

Tools used	Purpose	Outputs
**Kaizen**	Brainstorming session to gather inputs on the issues identified as per the RQs and analyse the problems identified. Analysis of Pareto's and histograms related to process and maintenance data	Identification of key focus areas for improvement and any points of change required.
**Equipment Criticality Analysis**	A multi-discipline team was drawn from within the organisation where the personnel and expertise existed with specialist consultants and Subject matter experts (SMEs) at project stages. This exercise assesses the Consequence of failure for each asset and assesses the Environmental Health and Safety, Business, Quality and Operational Availability risk, assigning a score to each criterion. The likelihood would be determined using a combination of Asset Maintainability and Probability of Failure.	A risk table and criticality matrix was developed that could be used to assign an overall risk ranking for each asset and equipment piece in order to prioritise preventative maintenance.
**Maintenance Optimisation** **Predictive & Preventative**	Maintenance Optimisation is the process that would balance the maintenance requirements legislative, economic, technical and the resources required to carry out the maintenance program. The purpose of the maintenance optimisation process would be to select the appropriate maintenance technique for each piece of equipment within a system.	This identifies the periodicity that the maintenance technique should be conducted to achieve regulatory requirements, and maintenance targets concerning safety, equipment reliability, and system availability.
Maintenance Optimisation Corrective Maintenance (CM) Optimisation	CM work orders should be prioritised by the engineering SME based on the Criticality Ranking to ensure Maintenance Resources are focused on the most critical equipment to the business. Considering that the design was given to the Sites Operational Schedule to effectively plan corrective maintenance, while supporting the site where possible to achieve its production targets.	A CM Prioritisation Ranking 7 strategy matrix
Failure Mode & Effect Analysis (FMEA)	FMEA studies were facilitated by the project team. This task involved key operational, process safety and engineering personnel in a more detailed analysis of the most critical assets identified during the Equipment Criticality Analysis process. The asset failure modes and their effects were analysed.	The root cause of failure was established, and the warning effects identified.
Reliability Centred Maintenance (RCM)	RCM is the process that would ensure maintenance tasks are performed in an efficient, cost-effective, reliable, and safe manner, minimising impact on Production. Maintenance tasks can be preventive, predictive, or involve non-destructive inspections to identify or monitor flaws. The purpose of RCM would be to ensure maintenance and inspection tasks are centred around improving the reliability and safety of equipment	A Reliability Centred Maintenance process

#### 3.2.4 Design

A strategy for Maintenance Optimisation: Preventive and Predictive Maintenance Optimisation w brainstormed and designed. In this stage it was identified the periodicity that the maintenance technique should be conducted to achieve regulatory requirements, and maintenance targets concerning safety, equipment reliability, and system availability.

This design focused on increasing equipment reliability by utilising two essential elements, Effectiveness and Efficiency. The effectiveness element will be aimed at avoiding critical equipment failures and associated costs. This would be accomplished through a risk-based approach utilising outputs from the Equipment Criticality Analysis, ensuring the optimal mixture of maintenance/reliability techniques incorporating Preventative Maintenance (PM), Predictive Maintenance (PdM), Reliability Centred Maintenance (RCM), Condition Based Monitoring (CBM) and Total Productive Maintenance (TPM). Firstly Table 3 and Table 4 was utilised to assess the asset maintainability and risk criteria.

**Table 3:**
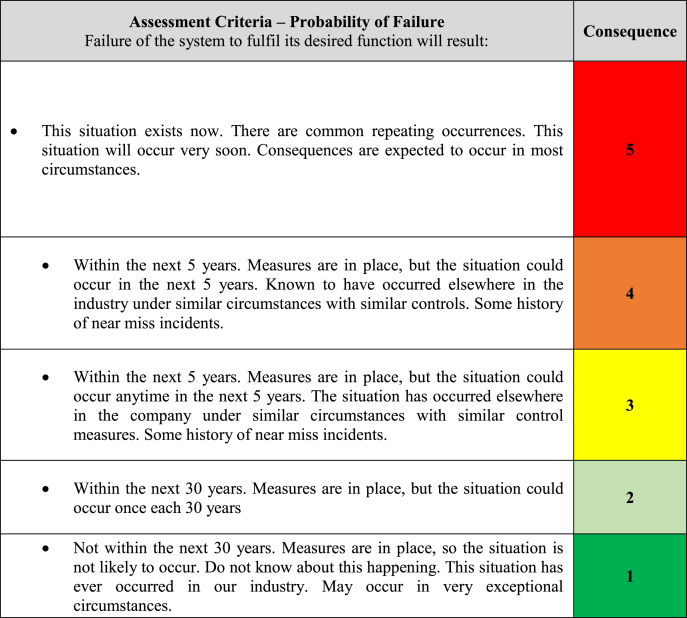
Criticality Equipment and Asset Maintainability (Source: Authors own).

**Table 4:**
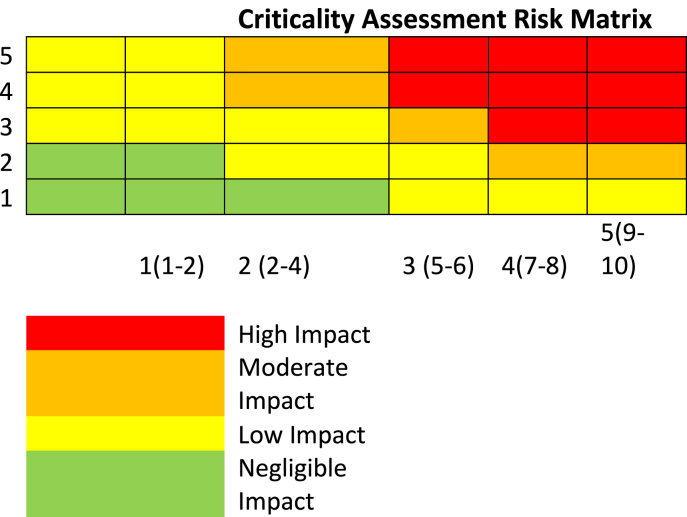
Risk Matrix (Source: Authors own).

The efficiency element would be targeted at clarifying maintenance work processes, roles, and responsibilities. This would ensure that the competent person or external vendor performs the correct Maintenance procedures at the right time. The subsequent Maintenance Optimisation strategy is highlighted in Table 5.

**Table 5:** Maintenance Optimisation (Source: Authors own).

Criticality Ranking	Strategy
A: High Impact	Detailed FMEA study to establish and implement robust maintenance/TPM plan.
B: Moderate Impact	Predictive, Preventive maintenance and RCM tasks. Consideration of FMEA.
C: Low Impact	Standard Preventive Maintenance Routines/Run to Fail (where appropriate)
D: Negligible Impact	Run to Fail, corrective maintenance only

Next a Corrective Maintenance (CM) Optimisation table was developed to aid CM Prioritisation, which guides how to use outputs from Reliability Criticality Analysis (Table 6).

**Table 6:** CM Prioritisation (Source: Authors own).

Criticality Ranking	Strategy
A: High Impact	Break into plan, repair to be prioritised urgently
B: Moderate Impact	Repairs to be prioritised by Engineering Planner
C: Low Impact	Repairs are to be planned accordingly by Engineering Planner
**D: Negligible Impact**	Low priority

#### 3.2.5 Verify

Utilising all of the aforementioned tools enabled the design and understanding of the TPFM new process and implementing and verification of the proposed design. All improvement and maintenance opportunities were logged within the project documentation for review by the team. A priority matrix scoring was utilised to select impactful opportunities for project inclusion. The results of the analyse phase would be incorporated into the outputs of the program and developed for the asset under review.

An outline of the results as validation and verification of the design framework for TPM are discussed in Section 4. The TPM techniques implemented minimised the requirement for impactful maintenance, supporting the site to achieve its production objectives while utilising the operator's equipment knowledge to proactively identify early indicators of equipment degradation. Continuous Improvement is critical to ensure that TPM and other reliability initiatives evolve driven by key stakeholders' feedback to achieve incremental improvements to achieve sustained reliability excellence. The design was verified as effective and the project team will develop and implement key performance metrics that the site will measure, track, and report.

## 4 Results

### 4.1 Define

As outlined previously a project charter was developed by the Project team defined the problem/aim, framed the scope of the overall project, and ensured that the project objectives were identified. The CTQs listed by the stakeholders were incorporated into the project definition and scope. The achievement of the CTQs helps the site focus the maintenance plans on the most critical aspects of the equipment and processes. In achieving the CTQs the business would expect to see an overall performance improvement. The Pilot Program was proposed, and a decision was made to implement the TPMR program on one singular asset [51].

The following criteria was utilised in the asset selection process, it must have.

1. Criticality of equipment – Importance to site
2. Process performance – Downtime (D/T) and Uptime
3. Functionality/Bottlenecks – Impact of availability
4. Machine Novelty (Uniqueness) – Number of units in operation

The selected process for optimisation via the methodology was a centrifugal process (CE504) within one of the API processing plant. The centrifuge is the solids/liquids separation device in an API plant. Slurry mixtures are fed into the machine via an input nozzle that feeds into the machine body. The product emitted from the centrifuge can be directly packaged into drums. Periodic equipment cleaning is required as per the cleaning validation requirements onsite. Inter-campaign cleaning (ICC) is required when changing from intermediate manufacture to API. Cleaning is required to enable maintenance tasks to be completed as per the site CMMS and additional cleaning is required after the maintenance work before commencing operations. The cleaning methods used on the CE504 centrifuge are a combination of clean-in-place (CIP) and manual cleaning. Additional maintenance activities are required to prepare the equipment for cleaning include strip down and rebuilding after the cleaning.

### 4.2 Measure

A data collection plan was implemented to capture information relevant to the centrifuge process. The equipment process data was collaboratively analysed to enable the current state of the process to be visualised and evaluated.

- Pareto graphs were utilised to define the most impactful downtimes in pareto form, these non-value add, and corrective maintenance activities needed to be reduced or removed to improve the overall process performance
- The planned maintenance tasks and maintenance hours required per year were identified and listed for analysis.
- The processing batch runtime and batch master batch record (MBR) data reviewed
- The process equipment cleaning times were listed for review. This cleaning is detailed as inter-campaign cleaning (ICC), post-campaign cleaning (PCC) and after-maintenance cleaning (MT).

### 4.3 Analyse & design

Lessons learned from across the global network were shared and included in the TPMR deployment strategy. Success factors for a program implementation were identified as leadership support, and organizations readiness to change. TPM Training was supported and a TPM support team was assigned to monitor and manage.

#### 4.3.1 equipment criticality analysis

Equipment Criticality Analysis was facilitated using the TPMR template a partial snapshot of which is shown in Table 7 Equipment Criticality Analysis Ranking. The Objective was to assess the Consequence of the failure of equipment and components of the centrifuge process CE504 assessing Environmental Health and Safety, Business, Quality and Operational Availability risk, and assigning a score to the asset using the highest identified risk ranking. The likelihood was determined using a combination of Asset Maintainability and Probability of Failure. The overall risk ranking was a combination of the highest assigned Consequence of failure and the Likelihood.

**Table 7:**
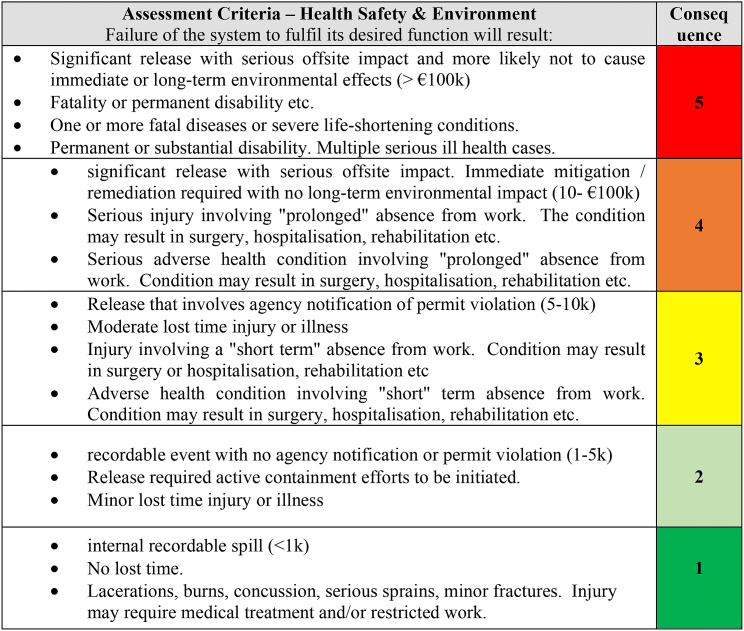
Equipment Criticality Analysis Ranking (partial snapshot) (Source: Authors own).

#### 4.3.2 Establish plant inventory and review plant availability

CE504 was identified as a critical asset and an FMEA was required. P&ID diagrams, Operational & Maintenance Manuals and Validation Files were studied. Operational logs were examined, and a tour of the plant was organised. Using this data and information gathered from the subject matter experts (SME) personnel a pareto graph and a reliability block diagram (RBD) was drawn up. The Pareto graph showed the number of operational failures affecting the system and analysis of the maintenance CMMS corrective work orders. The Reliability Block Diagrams (RBDs) were utilised to help identify critical plants and to show the availability of connections between equipment and systems. This enabled the project team to develop a better understanding of the equipment and system. A Failure Mode & Effects Analysis (FMEA) was utilised involving key operational, process safety and engineering personnel in a more detailed analysis of the most critical assets identified.

The asset failure modes and their effects were analysed and actions generated (Table 8). The root cause of failure was established, and the warning effects were identified.

**Table 8:** Key actions taken.

1. Training	Training was carried out with the key operational and engineering personnel who would be involved with the TPM study and program. This ensured that everybody was fully familiar with the overall process and the terminology, but also provides staff with a feeling of buy-in into the overall program
2. Determine the Optimum Maintenance Task	The characteristics of each failure mode identified in the FMEA were then studied to determine the consequence of failure and the failure distribution. This information was then used to determine the optimum task type required as per the following • Condition Monitoring • Scheduled Inspection • Scheduled Maintenance • Design Out • Fix On-Failure
3. Develop the Optimised Maintenance Plan	The project team enabled collective equipment ownership through the project methodology and teamwork with new recommended maintenance actions. They were compiled into the Optimised Maintenance Plan; key aspects of the plan were. • Operator Asset Care and Autonomous Maintenance tasks developed for plant operator execution. • Service Level Agreements improvements to support condition-based equipment monitoring. • Remote monitoring of assets as non-invasive maintenance tasks. • Pictorial Maintenance Work Instructions developed to support work execution on the plant. • Maintenance task frequencies extended because of improved CBM and RCM initiatives.

### 4.4 Overall Equipment Efficiency results

Equipment efficiency and effectiveness is the measure of the value added to production through the equipment. The pilot program and verify stage of DFLSS addressed repetitive corrective maintenance across all shifts. Target failures were identified from the shift and 12 month pareto graphs of equipment downtime. The top three failures 1. O2 Analyser, 2. Door locking and 3. Lubrication oil was reduced with a maintenance overhaul of the door assembly and vendor training on the operation and repair of the O2 analyser.

The top three downtime modes have been reduced from 151 h to 10 h in the 4 months post the TPMR introduction. The results were achieved by implementing the correct maintenance, the correct training and by introducing TPMR methodology. These results represent a 70 % reduction in corrective maintenance downtime on one asset CE504 alone.

The maintenance optimisation process reduced the frequency and requirement for impactful maintenance tasks as illustrated by Table 9. The CE504 planned maintenance duration was reduced from 67 h to 45 h because of the TPMR study. This equated to a 33 % reduction in PM time.

**Table 9:**
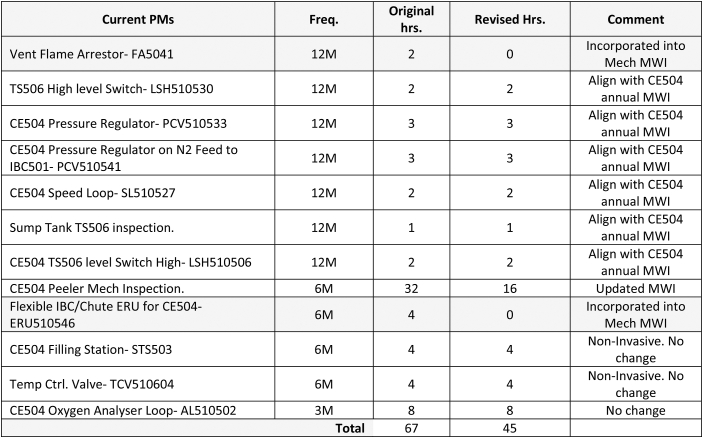
Maintenance Plan CE504 Rationalised (PM Hours per year).

The optimised maintenance plan enabled a reduction in campaign cleaning time, maintenance cleaning time and maintenance strip-down and reassembly requirements, as illustrated by Table 10. It is demonstrated that the requirement to stop and clean the centrifuge CE504 was a dominant negative impact on equipment availability identified in this research analysis.

**Table 10:**
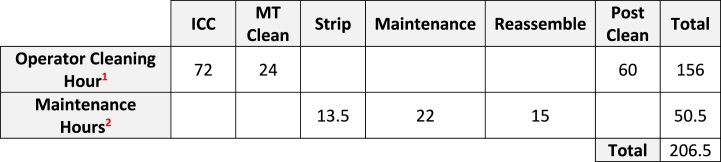
Reduction in Equipment Access Hours (Returned Availability).

In the implementation of the TPMR methodology within this research pilot program there is a reduction of 206.5 h. This 206.5 h is available to operations to manufacture and is classified as increased availability.

The result of introducing autonomous maintenance, 5S and RCM has enabled 206.5 h of equipment availability to be made available to Operations. This increase in availability is a result of amalgamating the six monthly and twelve-monthly maintenance tasks into one annual maintenance intervention. The introduction of TPMR discussed in this research methodology has enabled a positive impact on the OEE for centrifuge CE504 by identifying and addressing repetitive breakdown failures. In the introduction of TPMR, the early identification of potential failures has been enabled via operator care work instruction and condition monitoring technology tracking and monitoring equipment remotely.

The reliability-centred review completed in this TPMR research has enabled the maintenance techniques selected to mitigate against probable failure modes, enabled impactful planned maintenance to be amalgamated into an annual task and so removed the additional equipment cleaning requirements for Maintenance task execution.

Extrapolating on the results from Table 11 means that new figures based on 100 % utilisation of the newly calculate availability of 206.5 h equate to:

- A savings of 206.5 h Availability equates to an increase in 3.56 batches per year.
- The average batch yield is 1050 kg, yielding a realised batch yield is 1050 × 3.56 = 3734 kg per year.
- 1 kg of product has a value of €146.00, Added Product Value = €545,748 per year.

**Table 11:**
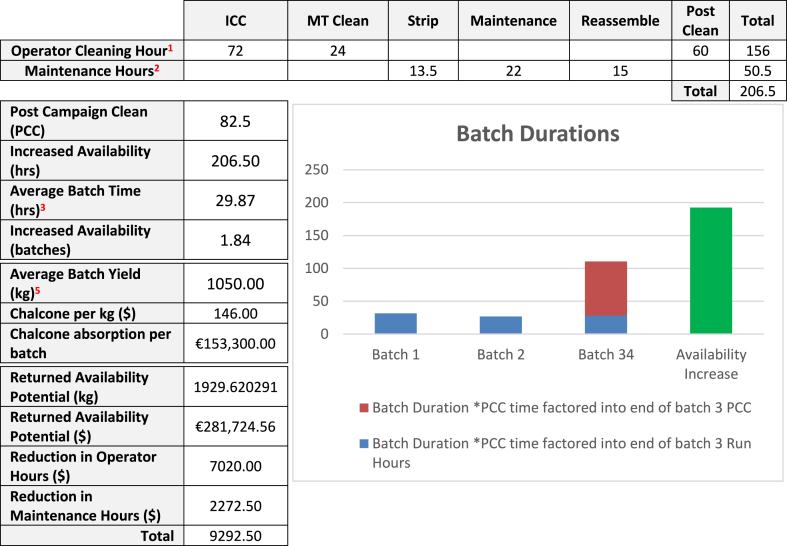
TPMR Return on Investment Calculations.

A well laid out work area can motivate employees to do good work and to maintain the standard. 5S is a Lean management tool and it is useful in eliminating clutter and waste within the workplace [61]*.* The centrifugal processing equipment will be maintained to 5S standards enabling the autonomous maintenance tasks to be executed with tools and equipment available in the area. The 5S program will be monitored via GEMBA and continuous improvements can be made to the work area and the process under the organizations OpEx program. Utilising 5S will build a self-directed work team, foster production equipment ownership, and increase asset utilisation [22].

**Fig. 1:**
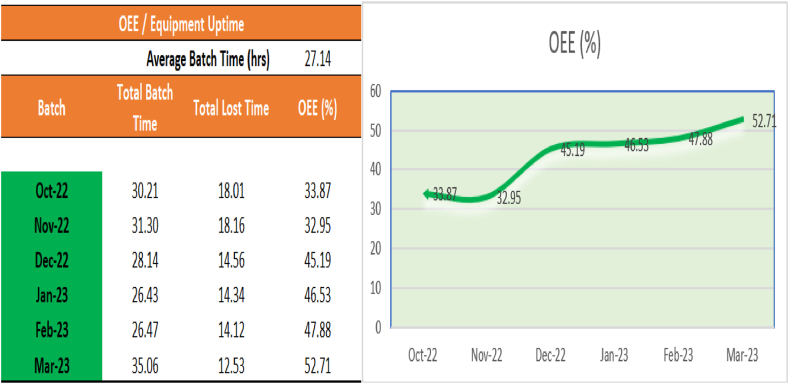
CE504 OEE KPI dashboard.

Managers use Key Performance Indicators (KPIs) to track manufacturing and business progress, review improvements and identifying potential issues. KPIs can be used to enable decision making and they assist employees making processing decisions. KPIs are defined as a set of measures that focus on the main critical activities can be defined as the core of performance monitoring [62]**.** The TPMR program for CE504 KPIs will be monitored and form a standard part of the site performance management meetings, as illustrated in Fig. 1 CE504 OEE KPI Dashboard. This will ensure that monitoring of the program and the process can be completed to ensure continued success.

### 4.5 Sustainability via a TPM framework and steering committee

Delivering the TPMR change initiative is not complete without building a communication platform and a monitoring steering committee to develop the change across other assets.

**Fig. 2:**
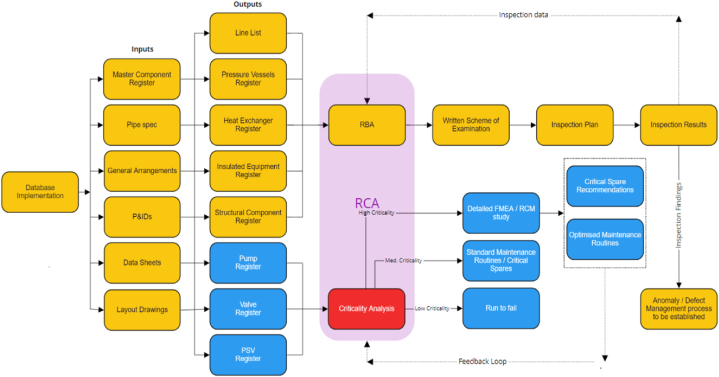
TPMR framework.

At Company ‘X’ there is a global platform that reviews the business framework scoring for sites, the actions planned to improve the process and an opportunity to share success stories via A3 project reporting. The site TPM team will prepare performance data for the meeting and attend weekly TPM steering committee meetings and so building on the program's success. The weekly meeting will enable other Lean initiatives to be considered for inclusion based on the 5S and KPI dashboards scoring reviews. The additional pillars of TPM can be reenforced through this steering committee. The TPMR framework is graphically represented as in Fig. 2.

## 5 Discussion

This research presents a new TPMR framework model for use at Company ‘X's API facility.

The research has developed a maintenance strategy program and enabled the development of a maintenance model linking Asset Care and Reliability Evaluation within a TPM program (RQ1) as well as enabling OEE improvement(RQ2).

This research has demonstrated the importance of collaboratively assessing asset reliability and performance at a manufacturing plant when developing new maintenance strategies. The TPMR framework was adopted and piloted at the API facility. The maintenance practices selected in this program are aligned with RCM, the eight pillars of TPM and the Regulatory Maintenance requirements of ISO 14224 to ensure industry robustness. Company ‘X's API plant is classified as a hazardous environments industry so utilises the data collection within the ISO 14224 standard to improve asset reliability, safety, and maintainability.

This TPMR framework and the Centrifugal pilot program took 12 months to develop and deploy. The actualised framework is now transferable across assets and equipment. A TPMR standard has been developed. In the utilisation of this new TPMR model similar time and financial results would be expected within 3 months of a new asset program deployment. The practical implication of this research is that the original calculated OEE of the asset CE504 was 32%. The new calculated OEE after the TPMR program is 53%. The TPMR program increased OEE and Asset Availability of 21% and 206.5 h respectively. The theoretical implication of the research is that Company ‘X’ operates four centrifugal processes across the API site. The equipment has identical technology and maintenance plans, based on the results obtained in this project the same TPMR program could be implemented on these assets. The potential output of these programs could yield an additional 618 h of centrifugal equipment availability over 12 months. Therefore, this research could increase the site centrifugal availability by 824.5 h collectively. This equates to an increase of 4.9 weeks of production capacity.

The TPMR model has attained Leadership support having documented results. This was achieved by a collaborative site effort where all team members input into all DFLSS project phases. In the execution of this research, it has been observed how leadership support for a TPM implementation initiative and collaborative departmental support has a positive impact on the site performance and capabilities [54]. Continuous Improvement is critical to ensure that TPM and site Reliability Objectives evolve driven by key stakeholders’ feedback to achieve incremental improvements to achieve sustained business excellence [55]. Implementation of a TPM program creates shared responsibility for equipment that encourages greater involvement by all stakeholders -thus increasing productivity and up time, reducing cycle times, and eliminating defects*,* [63]. TPM was not just a technology initiative but also is a change initiative, with strong cultural aspects of deployment.

The output of the literature review states that the implementation of an effective TPM program can affect manufacturing business performance positively and the results of this study corelate with the literature [16,52,59]. This case study article outlines the steps involved in developing an implementation plan for TPMR, including assessing the current maintenance practices, identifying areas for improvement, and creating a detailed implementation timeline. This research contributes to the literature by evaluating asset reliability with TPM and so goes beyond a standard TPM approach. The data analysis was completed using the DFLSS method where the program results realised a 33 % reduction in planned maintenance activities, cleaning for maintenance was reduced by 50 %, centrifuge OEE increased by 20 % and plant availability increased by two hundred and 6 h. Similar studies has discussed the importance of maintenance in OEE optimisation [15,64]. The integration of TPM and reliability is recommended to improve the manufacturing operations of an API plant.

## 6 Conclusion

The TPMR program implemented in this research was validated by the result attained. This research identified the benefits of TPM implementation for organizations, including improved equipment reliability, reduced maintenance costs, increased product quality and increased productivity (RQ1). This new model delivered additional plant availability and an increase in the plant OEE *(*RQ2*).* The implications of this study are to demonstrate to organizations that TPMR is an evolution of TPM and provides greater business benefits as it improves productivity. The study demonstrates that a TPMR framework can replace a standard maintenance strategy. This research has implications for organizations to significantly improve their business performance utilising the methodology detailed. Within the pharmaceutical industry in particular TPMR concepts and philosophy can be successfully applied to achieve manufacturing performance improvements within the extremely competitive API Manufacturing environment. From an academic implications viewpoint this pilot program unequivocally demonstrates that a TPMR implementation program can improve an API manufacturing plant's equipment availability and OEE and adds to the literature on TPMR and OEE. The study also has implications for the attainment of TQM by demonstrating that TQM and TPM can be realised with Lean practices.

Recommendations for additional consideration in future research is in removing the human interaction from equipment maintenance. As Industry 4.0 (I4.0), Artificial Intelligence (AI) and Data Analytics (DA) are advancing in capabilities, future research will investigate the integration of Lean and Six Sigma methodologies in conjunction with the digital transformation towards achieving strategic business advantage.

## Data availability

Data will be made available on request.

## Ethics declarations

Informed consent was not required for this study as there were no interviews with people nor were there vulnerable populations involved in the study.

## CRediT authorship contribution statement

**Noel Shannon:** Conceptualization, Data curation, Project administration, Writing – original draft. **Anna Trubetskaya:** Resources, Software, Writing – original draft, Writing – review & editing. **Javed Iqbal:** Data curation, Supervision, Visualization. **Olivia McDermott:** Conceptualization, Methodology, Project administration, Supervision, Writing – original draft, Writing – review & editing.

## Declaration of competing interest

The authors declare that they have no known competing financial interests or personal relationships that could have appeared to influence the work reported in this paper.
